# Plasma biomarker for detection of early stage pancreatic cancer and risk factors for pancreatic malignancy using antibodies for apolipoprotein-AII isoforms

**DOI:** 10.1038/srep15921

**Published:** 2015-11-09

**Authors:** Kazufumi Honda, Michimoto Kobayashi, Takuji Okusaka, Jo Ann Rinaudo, Ying Huang, Tracey Marsh, Mitsuaki Sanada, Yoshiyuki Sasajima, Shoji Nakamori, Masashi Shimahara, Takaaki Ueno, Akihiko Tsuchida, Naohiro Sata, Tatsuya Ioka, Yohichi Yasunami, Tomoo Kosuge, Nami Miura, Masahiro Kamita, Takako Sakamoto, Hirokazu Shoji, Giman Jung, Sudhir Srivastava, Tesshi Yamada

**Affiliations:** 1 Division of Chemotherapy and Clinical Research, National Cancer Center Research Institute, Tokyo 104-0045, Japan; 2 Toray Industries, Inc., New Frontiers Research Labs, Kanagawa 248-8555, Japan; 3 Hepatobiliary and Pancreatic Oncology Division, National Cancer Center Hospital, Tokyo 104-0045, Japan; 4 National Cancer Institute, Division of Cancer Prevention, Rockville, MD 20852, USA; 5 Public Health Sciences Division, Fred Hutchinson Cancer Research Center, Seattle 98109-1024, WA, USA; 6 Department of Surgery, Osaka National Hospital, National Hospital Organization, Osaka 540-0006, Japan; 7 Department of Oral Surgery, Osaka Medical College, Osaka 569-8686, Japan; 8 Department of Gastrointestinal and Pediatric Surgery, Tokyo Medical University, Tokyo 160-0023, Japan; 9 Department of Surgery, Jichi Medical University, Tochigi 329-0498, Japan; 10 Department of Hepatobiliary and Pancreatic Oncology, Osaka Medical Center for Cancer and Cardiovascular Diseases, Osaka 537-0025, Japan; 11 Department of Regenerative Medicine and Transplantation, Fukuoka University Faculty of Medicine, Fukuoka 814-0018, Japan; 12 Hepatobiliary and Pancreatic Surgery Division, National Cancer Center Hospital, Tokyo 104-0045, Japan; 13 Japan Agency for Medical Research and Development (AMED) CREST, Tokyo 100-0004, Japan

## Abstract

We recently reported that circulating apolipoprotein AII (apoAII) isoforms apoAII-ATQ/AT (C-terminal truncations of the apoAII homo-dimer) decline significantly in pancreatic cancer and thus might serve as plasma biomarkers for the early detection of this disease. We report here the development of novel enzyme-linked immunosorbent assays (ELISAs) for measurement of apoAII-ATQ/AT and their clinical applicability for early detection of pancreatic cancer. Plasma and serum concentrations of apoAII-ATQ/AT were measured in three independent cohorts, which comprised healthy control subjects and patients with pancreatic cancer and gastroenterologic diseases (n = 1156). These cohorts included 151 cases of stage I/II pancreatic cancer. ApoAII-ATQ/AT not only distinguished the early stages of pancreatic cancer from healthy controls but also identified patients at high risk for pancreatic malignancy. AUC values of apoAII-ATQ/AT to detect early stage pancreatic cancer were higher than those of CA19–9 in all independent cohorts. ApoAII-ATQ/AT is a potential biomarker for screening patients for the early stage of pancreatic cancer and identifying patients at risk for pancreatic malignancy (161 words).

Pancreatic cancer is one of the most lethal solid malignant tumors. To decrease the mortality of pancreatic cancer, efficient screening methods that will enable detection of the early stage of the disease and the identification of the precancerous lesions that are thought to be risk factors for pancreatic malignancy are needed^1^,^2^. Plasma/serum biomarkers for the early detection of pancreatic cancer would be useful clinical tools for screening patients in order to identify those who should undergo a second screening using stricter diagnostic modalities that can detect pancreatic dysfunction before imaging^3^.

We recently reported the results of a mass spectrometry (MS)-based proteomic analysis, which showed that the levels of five circulating isoforms of apolipoprotein-AII (apoAII), including two novel isoforms, are significantly different in the plasma of patients with invasive ductal adenocarcinoma of the pancreas (IDACP) relative to healthy controls^4^,^5^. The five circulating apoAII-isoforms are characterized by the truncation of varying numbers of amino acids from the C-terminus of the apoAII homo-dimer. The isoforms were designated apoAII-ATQ/ATQ (apoAII-1, 17,380 Da) (the descriptions of -ATQ/ATQ etc. showed that each had the C-terminal sequence of an apo-AII isoform), apoAII-ATQ/AT (apoAII-2, 17,252 Da), apoAII-AT/AT (apoAII-3, 17,124 Da), apoAII-AT/A (apoAII-4, 17,023 Da), and apoAII-A/A (apoAII-5, 16,922 Da) (Supplemental Figure 1)^5^. The circulating isoforms could be distinguished according to differences in molecular weight as determined by matrix-assisted laser desorption/ionization-mass spectrometry (MALDI-MS)^5^.

We previously reported the development of a novel and sophisticated MALDI-MS method for the semi-quantitative measurement of the levels of apoAII-isoforms in plasma. This MALDI-MS method was used to analyze more than 1,300 plasma/serum samples collected from patients at multiple medical institutions in Japan and Germany, in a previous study^5^. These analyses showed a statistically significant decrease in the level of apoAII-ATQ/AT in plasma and serum of IDACP patients compared with healthy controls from four independent cohorts^5^. These results suggested that apoAII-ATQ/AT would be good candidate plasma biomarkers for use in diagnosing early stage pancreatic cancer^5^,^6^, unlike other isoforms such as apoAII-ATQ/ATQ, -AT/AT, AT/A and A/A^5^.

However, several factors impeded the clinical application of our MALDI-MS–based method for the measurement of apoAII-ATQ/AT. In this study, we developed novel sandwich ELISAs for the measurement of apoAII-isoforms in clinical samples. Our sandwich ELISAs provide for robust and rapid analysis of apoAII-isoforms. We evaluated the assays by measuring the plasma levels of apoAII-isoforms in samples from patients with pancreatic cancer and pancreatic disorders, including precancerous lesions and various malignant diseases of other organs, and we then compared the results with those of healthy controls.

The Early Detection Research Network (EDRN), an initiative of the National Cancer Institute (NCI), is a consortium of institutions with the goal of accelerating the translation of biomarker information into clinical applications for the early detection of cancer (http://edrn.nci.nih.gov). The objectives of the EDRN include the development and testing of promising biomarkers or technologies for early detection of cancer and the evaluation of promising, analytically proven biomarkers or technologies. The EDRN has reference sets available for validating promising biomarkers for the early detection of cancer. The Japanese team applied for and received the pancreatic cancer reference set to validate the apoAII-isoforms biomarker for early detection of this cancer.

## Results

### Establishment of ELISAs for measuring apoAII-isoforms

We established a novel anti-human apoAII-AT rabbit polyclonal antibody and an anti-human apoAII-ATQ mouse monoclonal antibody. We then developed novel sandwich ELISAs for measuring these apoAII-isoforms using the newly established antibodies.

**Figure 1:**
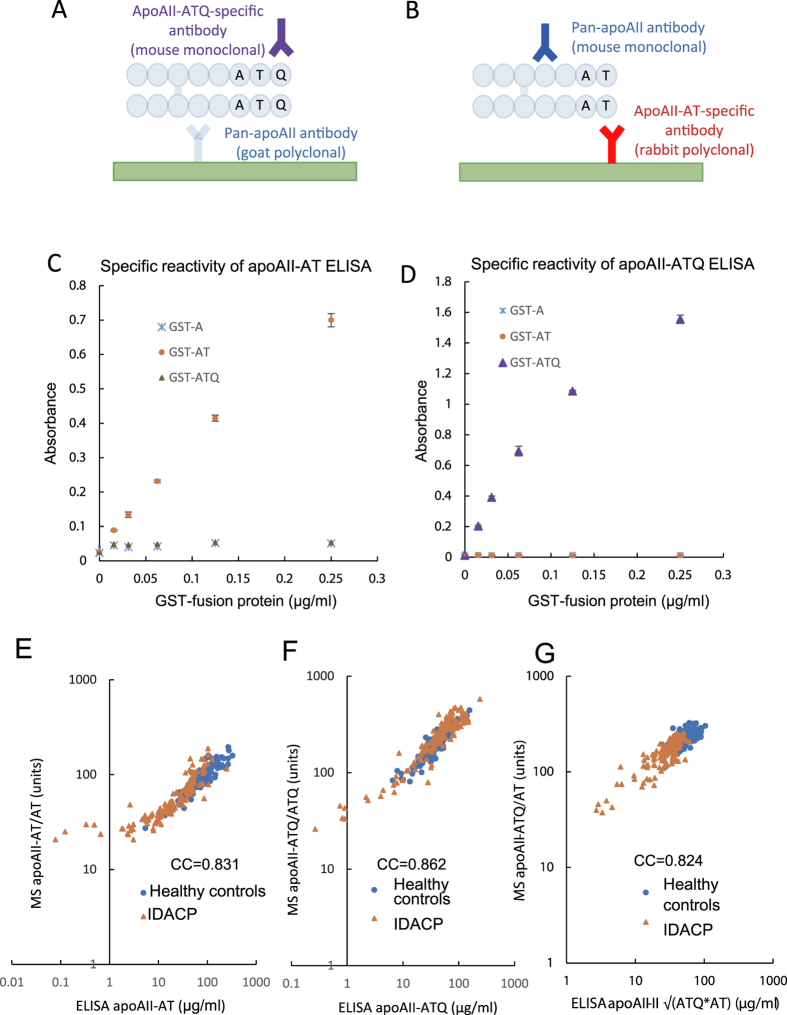
ApoAII-isoform ELISAs, specificity of anti-apoAII-isoform antibodies, and correlation between ELISA- and MS-based results. Details of the (**A**) apoAII-ATQ and (**B**) apoAII-AT ELISAs. The specific reactivity of ELISA (apoAII-AT ELISA (**C**) and apoAII-ATQ ELISA (**D**)) in regard to GST-fusion-apoAII-A (blue asterisks), -apoAII-AT (orange circles), and -apoAII-ATQ (purple triangles). Correlation between results of MS-based assay and ELISAs (**E–G**). Two-dimensional scatter graph of apoAII-AT/AT as determined by MS-based assay (MS apoAII-AT/AT) and apoAII-AT as determined by ELISA (ELISA apoAII-AT) (**E**). Two-dimensional scatter graph of MS apoAII-ATQ/ATQ and ELISA apoAII-ATQ (**F**). Two-dimensional scatter graph MS apoAII-ATQ/AT and ELISA apoAII 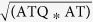 (ELISA apoAII-ATQ/AT) (**G**). Healthy controls (blue circles) and invasive ductal adenocarcinoma of the pancreas (IDACP, orange triangles).

Schematic illustrations of the sandwich ELISAs for measuring apoAII-ATQ and -AT are shown in Fig. 1A,B. For the apoAII-ATQ ELISA, the pan-apoAII goat polyclonal antibody was coated on the well surfaces of the microtiter plate as the capture antibody, and the apoAII-ATQ–specific mouse monoclonal antibody was used for detection (Fig. 1A). For the apoAII-AT ELISA, the apoAII-AT–specific rabbit polyclonal antibody was coated on the microtiter plate well surfaces as the capture antibody, and the pan-apoAII mouse monoclonal antibody was used for detection (Fig. 1B).

To confirm the specificity of each sandwich ELISA for the respective apoAII-isoforms, we assessed the cross reactivity of each assay using apoAII-ATQ and apoAII-AT that were fused to glutathione S-transferase (GST) proteins (Fig. 1C,D). Both sandwich ELISAs exhibited high specificity for the respective GST-apoAII fusion proteins. Moreover, no cross reactivity was observed.

**Figure 2:**
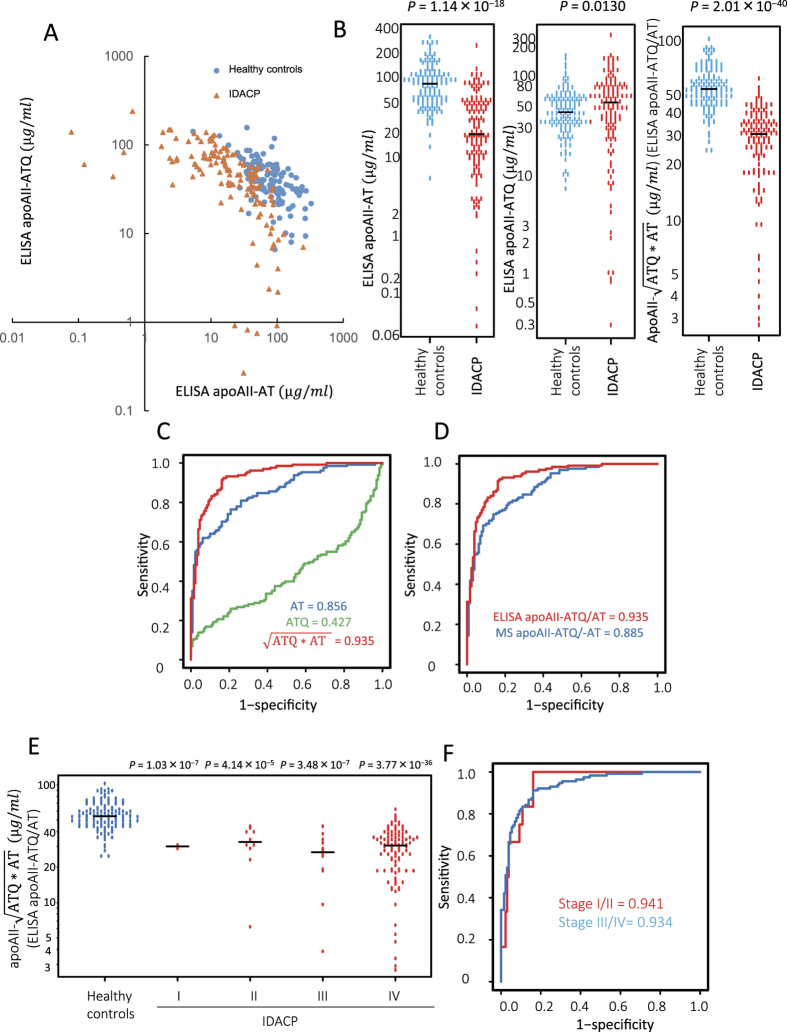
Distribution of apoAII-isoforms in healthy controls and IDACP patients, and ROC and AUC values for apoAII-isoforms for distinguishing IDACP patients from healthy controls in the single-institute cohort (cohort-1). (**A**)Two-dimensional scatter graph of apoAII-AT as determined by ELISA (ELISA apoAII-AT) and apoAII-ATQ as determined by ELISA (ELISA apoAII-ATQ), (healthy controls, blue circles; IDACP, orange triangles). (**B**) Distribution of each apoAII isoform in healthy controls (blue circles) and IDACP patients (red circles) (left graph, ELISA apoAII-AT; middle, ELISA apoAII-ATQ; right, ELISA apoAII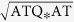; [ELISA-apoAII-ATQ/AT]), (P-value, Student’s t-test). (**C**) ROC and AUC values for ELISA apoAII-isoforms for distinguishing IDACP patients from healthy controls (blue line, ELISA apoAII-AT; green line, ELISA apoAII-ATQ; red line, ELISA apoAII-ATQ/AT). (**D**) ROC and AUC values for ELISA apoAII-ATQ/AT (red line, ELISA apoAII-ATQ/AT) and MS apoAII-ATQ/AT (blue line) for distinguishing IDACP patients from healthy controls. (**E**) The distribution of ELISA apoAII-ATQ/AT in healthy controls and in patients at each clinical stage of IDACP (P-value, Student’s t-test). (**F**) ROC and AUC values for ELISA apoAII-ATQ/AT for distinguishing patients at each clinical stage of IDACP from healthy controls (red line; stage-I/II, blue line; stage-III/IV).

To confirm the reliability of the sandwich ELISAs, we determined the correlation between the MS and ELISA results using plasma samples that had been previously analyzed by MS^5^. Plasma samples from age- and sex-matched healthy donors (n = 130) and IDACP patients (n = 131) (cohort-1) were collected at the National Cancer Center Central Hospital (NCCH) (Table 1). The samples of cohort-1 were comprised of 2 cases of stage-I IDACP, 10 cases of stage-II, 12 cases of stage-III, 102 cases of stage-IV, and 5 cases of unknown stage. There was high correlation between healthy controls and IDACP patients with respect to the results for apoAII-AT/AT obtained using MS and those obtained for apoAII-AT using the sandwich ELISA (Fig. 1E; correlation coefficient [CC] = 0.831) and for apoAII-ATQ/ATQ (MS) and apoAII-ATQ (sandwich ELISA) (Fig. 1F; CC = 0.862) (Supplemental Table 1). These data suggest that the results of the apoAII-AT sandwich ELISA are indicative of the level of plasma apoAII-AT/AT and that therefore, apoAII-AT could serve as a surrogate biomarker. The same holds true for the apoAII-ATQ sandwich ELISA, suggesting that apoAII-ATQ could also serve as a surrogate biomarker for the plasma level of apoAII-ATQ/ATQ. When we measured the plasma levels of five apoAII-isoforms by MS, we found that the concentrations of apoAII-ATQ/ATQ and apoAII-AT/AT were negatively correlated (Supplemental Figure 2). The level of apoAII-ATQ/AT in particular was closely associated with decreased levels of both apoAII-ATQ/ATQ and apoAII-AT/AT in IDACP patients and with decreases in the levels of either apoAII-ATQ/ATQ or apoAII-AT/AT (Supplemental Figure 2 and Fig. 2A).

When we examined the distributions of apoAII-ATQ/AT and other apoAII-isoforms in the MS data generated using samples obtained from IDACP patients of cohort-1, the isoforms seemed to distribute into two groups. ApoAII-ATQ/ATQ, which has a higher molecular weight than apoAII-ATQ/AT, was the dominant component in the first group, whereas the dominant components in the second group were apoAII-AT/AT, -AT/A, or -A/A, which are of lower molecular weight than apoAII-ATQ/AT (Supplemental Figure 3). These data suggest that cohort-1 was composed of two types of IDACP patients, one of which could be classified based on hyper-processing of the apoAII-homo-dimer (Supplemental Figure 3, brown arrow; Supplemental Figure 4, brown group) and the other based on hypo-processing of the apoAII homo-dimer (Supplemental Figure 3, purple arrow; Supplemental Figure 4, purple group; see also the Discussion). To define surrogate biomarker levels of apoAII-ATQ/AT, we developed a formula to calculate the concentration of apoAII-ATQ/AT (μg/ml) based on ELISA results for apoAII-ATQ and apoAII-AT (equation-1):

apoAII-ATQ/AT (μg/ml) =ELISA ApoAII 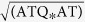


A high correlation was observed between the results for apoAII-ATQ/AT obtained by MS and those calculated using equation-1 (CC = 0.824; Fig. 1G). Based on this result, we defined a solution of equation-1 as the surrogate biomarker for apoAII-ATQ/AT.

### Clinical evaluation of apoAII-isoforms in cohort-1 patient samples

The expression levels of the apoAII-isoforms were examined by ELISA using plasma samples collected at the NCCH from cohort-1, which included healthy controls and patients with IDACP (Table 1). No statistically significant differences in sex and age were observed between healthy controls and IDACP patients in cohort-1. The levels of apoAII-AT and -ATQ in the plasma of patients with IDACP were inversely correlated (Fig. 2A). The average plasma level of apoAII-AT was significantly lower in IDACP patients compared with healthy controls (*P* = 1.14 × 10^−18^, Student’s t-test) (Fig. 2B, left panel). In contrast, the average plasma level of apoAII-ATQ was slightly but significantly higher in IDACP patients compared with healthy controls (*P* = 0.0130) (Fig. 2B, middle panel). Using equation-1, we estimated the level of apoAII-ATQ/AT based on the data for apoAII-ATQ and apoAII-AT. The concentration of apoAII-ATQ/AT in plasma samples from IDACP patients was significantly lower than that in samples from healthy controls (*P* = 2.01 × 10^−40^) (Fig. 2B, right panel).

Receiver operating characteristic (ROC) analyses revealed that the area under the curve (AUC) for apoAII-ATQ/AT determined by ELISA is 0.935 in cohort-1, a value higher than that for both apoAII-ATQ (AUC = 0.427) and apoAII-AT (AUC = 0.856) (Fig. 2C). The AUC for apoAII-ATQ/AT levels determined by ELISA was 0.935, whereas that for levels determined by MS analysis for cohort-1 was 0.885 (Fig. 2D).

The level of apoAII-ATQ/AT, as determined by ELISA, in samples from patients with different stages of IDACP was significantly lower compared with the level in healthy controls, and this significant difference was observed even in patients in the early stages of pancreatic cancer (i.e., stages-I and -II) (Fig. 2E). The AUC values of apoAII-ATQ/AT to distinguish patients with stage-I/II, and -III/IV IDACP from healthy controls were 0.941 and 0.934, respectively, in cohort-1 (Fig. 2F). We compared clinical efficiency between using the whole amount of apoAII and a specific amount of apoAII-ATQ/AT to detect IDACP using samples from cohort-1. The mean whole amount in healthy controls and IDACP was 72.9 μg/ml and 69.6 μg/ml, respectively. The AUC value of the whole amount of apoAII was 0.666 (Supplemental Figure 5A). The AUC values of the whole amount for stage-I/II, and stage-III/IV of IDACP were 0.667 and 0.661, respectively (Supplemental Figure 5B). The AUC value of apoAII-ATQ/AT, 0.935, was higher than that of the whole amount of apoAII.

### ELISA determination of apoAII-isoforms in samples from an independent multi-institution cohort (cohort-2) and comparison of ELISA results for apoAII-isoforms with those for existing pancreatic cancer biomarkers

**Figure 3:**
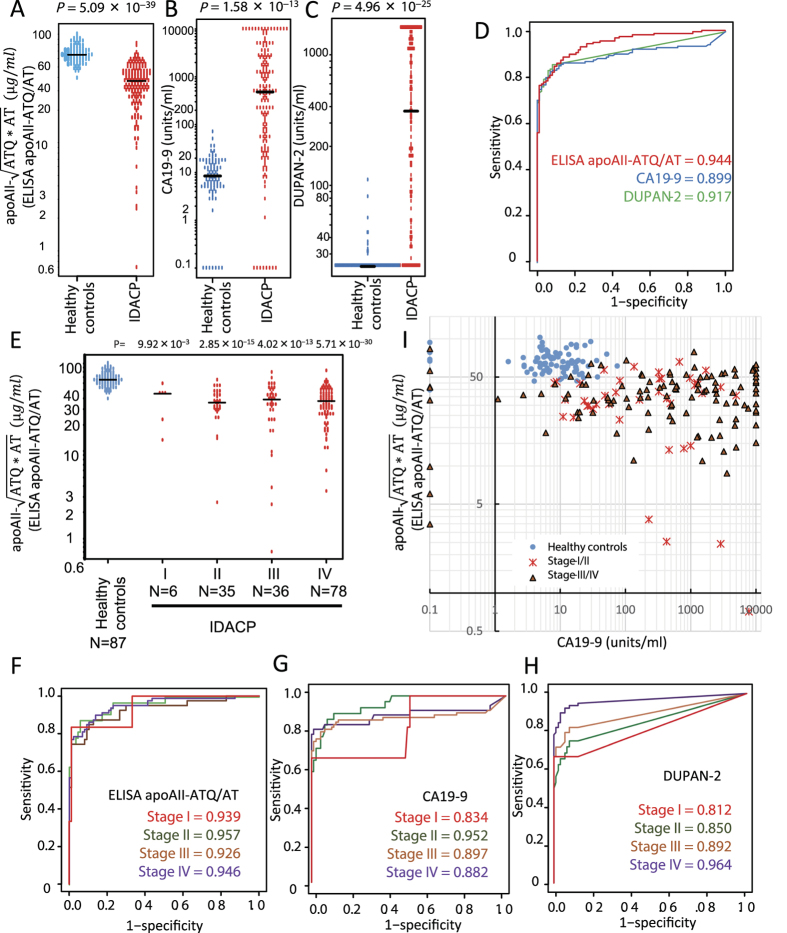
Distributions and AUC values for ELISA apoAII-ATQ/AT, CA19–9, and DUPAN-2 results and combination ELISA apoAII-ATQ/AT and CA19–9 analysis of samples from the multi-institution cohort (cohort-2). Distributions in healthy controls and IDACP patients of ELISA apoAII-ATQ/AT (**A**) CA19–9 (**B**), and DUPAN-2 (**C**) (blue circles, healthy controls; red circles, IDACP; black bars, medians. P-values, Student’s t-test). ROC curves and AUC values for ELISA apoAII-ATQ/AT (red line), CA19–9 (blue line), and DUPAN-2 (green line) (**D**). The distribution of ELISA apoAII-ATQ/AT in patients at each clinical stage of IDACP (**E**), (black bars, medians. P-values, Student’s t-test). ROC and AUC values for distinguishing patients at each clinical stage of IDACP (red line, stage-I; green line, stage-II; orange line, stage-III; purple line, stage-IV) from healthy controls by ELISA apoAII-ATQ/AT (**F**), CA19–9 (**G**), and DUPAN-2 (**H**). Combination ELISA apoAII-ATQ/AT and CA19–9 analysis (blue circles, healthy controls; red dots, stage-III IDACP; orange triangles, stage-III/IV IDACP) (**I**).

To validate the diagnostic accuracy of the apoAII-isoform ELISA for IDACP, the concentrations of apoAII-isoforms in samples obtained from an independent, multi-institution cohort involving seven medical institutes in Japan (cohort-2) were evaluated^5^,^6^. The plasma samples of cohort-2 were taken from healthy controls (n = 87) and patients with IDACP (n = 155), pancreatic disease other than IDACP (n = 57), cholangiocarcinoma (n = 26), duodenal carcinoma (n = 11), hepatocellular carcinoma (n = 12), esophageal carcinoma (n = 11), gastric carcinoma (n = 142), and colorectal carcinoma (n = 142). Of the patients with IDACP, there were 6 cases of stage-I, 35 cases of stage-II, 36 cases of stage-III, and 78 cases of stage-IV (Table 2). The level of apoAII-ATQ/AT in samples from IDACP patients was significantly lower than that in samples from healthy controls (Fig. 3A; Supplemental Table 2). The average plasma apoAII-ATQ/AT level in healthy controls was 66.7 μg/ml, compared with only 36.6 μg/ml in IDACP patients (*P* = 5.09 × 10^−39^, Student’s t-test). Statistically significant differences were also observed between healthy controls and IDACP patients in the levels of carbohydrate antigen 19–9 (CA19–9) (*P* = 1.58 × 10^−13^; Fig. 3B) and DUPAN-2 (*P* = 4.96 × 10^−25^; Fig. 3C). AUC values for apoAII-ATQ/AT, CA19–9, and DUPAN-2 for distinguishing IDACP patients from healthy controls were 0.944, 0.899, and 0.917, respectively (Fig. 3D). A decrease in the concentration of apoAII-ATQ/AT relative to the concentration in healthy controls was observed in samples of patients with stage-I and stage-II IDACP. The average apoAII-ATQ/AT concentrations in samples from healthy controls and patients with stages–I (n = 6), -II (n = 35), -III (n = 36), and –IV (n = 78) IDACP were significantly lower than those in healthy controls (66.7 μg/ml; P = 9.92 × 10^−3^, 38.8 μg/ml; 2.85 × 10^−15^, 36.7 μg/ml; 4.02 × 10^−13^, 36.8 μg/ml and 5.71 × 10^−30^, 36.4 μg/ml, respectively; Fig. 3E) (Table 2).

AUC values of apoAII-ATQ/AT to distinguish patients with stage-I, II, III, and IV IDACP from healthy controls were 0.939, 0.957, 0.926, and 0.946, respectively. AUC values for all stages were greater than 0.90 (Fig. 3F). On the other hand, AUC values for CA19–9 to distinguish patients with stage-I, II, III, and IV IDACP from healthy controls were 0.834, 0.952, 0.897, and 0.882, respectively (Fig. 3G). The AUC values for DUPAN-2 for distinguishing patients with stage-I, II, III, and IV IDACP from healthy controls were 0.812, 0.850, 0.892, and 0.964, respectively (Fig. 3H). Moreover, the AUC value for apoAII-ATQ/AT in patients with stage-I IDACP was higher than those for CA19–9 and DUPAN-2 in stage-I patients.

Analysis of the levels of both CA19–9 and apoAII-ATQ/AT by combination ELISA revealed a complementary association between CA19–9 and apoAII-ATQ/AT that increased the diagnostic accuracy in detecting the early stages of IDACP. We defined cut-off values for apoAII-ATQ/AT and CA19–9 as 46.3 μg/ml and 75 units/ml, respectively. In the apoAII-ATQ/AT and CA19–9 combination assay, we defined an apoAII-ATQ/AT level of less than 46.3 μg/ml and/or a CA19–9 level of more than 75 units/ml as IDACP. Based upon this definition, the sensitivity of the apoAII-ATQ/AT and CA19–9 combination assay for discriminating IDACP patients from healthy controls was 95.4%, and its specificity was 98.3% (Fig. 3I). The sensitivity of the combination assay for detecting stage-I IDACP was 100%, and the sensitivity for detecting stage-II was 97.1% (Fig. 3I). A cut-off value of 37 units/ml for CA19–9 is typically used in the general clinical field. In cohort-2, CA19–9 levels <37 units/ml were observed in 36 IDACP patients, whereas the apoAII-ATQ/AT ELISA identified 31 patients with CA19–9 levels <37 units/ml (Fig. 3I).

### Distribution of apoAII-isoforms in other gastroenterologic diseases

**Figure 4:**
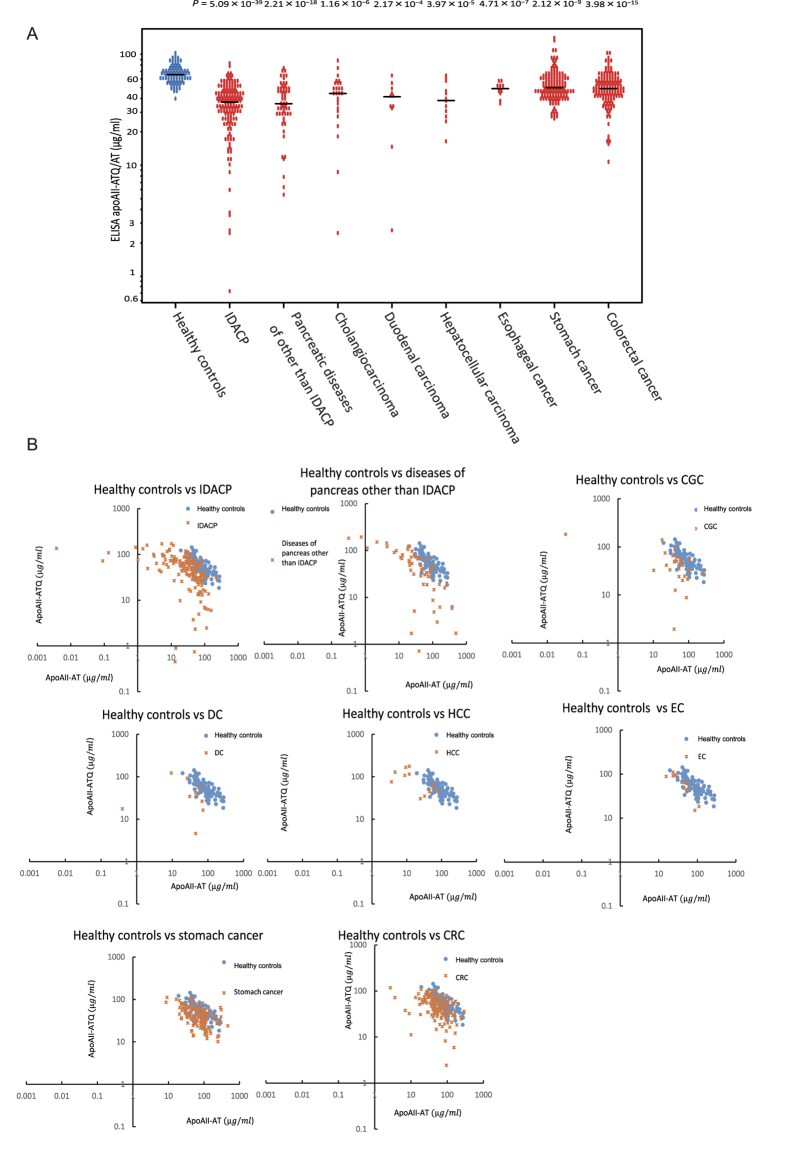
Distribution of ELISA apoAII-ATQ/AT results and two-dimensional scatter graphs of ELISA apoAII-ATQ and -AT results in patients with various gastroenterologic diseases in the multi-institution cohort (cohort-2). (**A**) Distributions of ELISA apoAII-ATQ/AT results in patients with various gastroenterologic diseases (blue circles, healthy controls; red circles, gastroenterologic diseases; black bars, medians. P-values, Student’s t-test). (**B**) Two-dimensional scatter graphs of ELISA apoAII-ATQ and -AT results in patients with various gastroenterologic diseases (blue circles, healthy controls; orange dots, gastroenterologic diseases; healthy, healthy controls; invasive ductal adenocarcinoma of the pancreas, IDACP; cholangiocarcinoma, CGC; duodenal carcinoma, DC; hepatocellular carcinoma, HCC; esophageal cancer, EC; colorectal cancer, CRC).

Figure 4A shows the distribution of apoAII-ATQ/AT in healthy controls and patients in cohort-2 with various other gastroenterologic diseases. The apoAII-ATQ/AT level was significantly lower in patients with IDACP (n = 155; average = 36.6 μg/ml; Student’s t-test, *P* = 5.09 × 10^−39^), pancreatic diseases other than IDACP (n = 57; 38.5 μg/ml; *P* = 2.21 × 10^−18^), cholangiocarcinoma (n = 26; 43.3 μg/ml; *P* = 1.16 × 10^−6^), duodenal carcinoma (n = 11; 37.7 μg/ml; *P* = 2.17 × 10^−4^), hepatocellular carcinoma (n = 12; 39.9 μg/ml; *P* = 3.97 × 10^−5^), esophageal carcinoma (n = 11; 48.0 μg/ml; *P* = 4.71 × 10^−7^), gastric cancer (n = 142; 53.8 μg/ml; *P* = 2.12 × 10^−9^), and colorectal cancer (n = 142; 50.4 μg/ml; *P* = 3.98 × 10^−15^), in comparison with healthy controls (n = 87; 66.7 μg/ml) (Fig. 4A and Supplemental Table 3). The decrease in apoAII-ATQ/AT relative to healthy controls was particularly dramatic in patients with IDACP, pancreatic diseases other than IDACP, cholangiocarcinoma, and duodenal carcinoma (Fig. 4A and Supplemental Table 3). Two-dimensional scatter graphs showing the levels of apoAII-ATQ and apoAII-AT in healthy controls and patients with other gastroenterologic diseases are shown in Fig. 4B. Substantially lower levels of apoAII-ATQ or -AT (<1.0 μg/ml) were observed in samples from patients with IDACP, pancreatic diseases other than IDACP, cholangiocarcinoma, and duodenal carcinoma (Fig. 4B).

To rule out a possible effect of jaundice on apoAII-isoforms, we calculated the CCs between apoAII-isoforms and total bilirubin in plasma of healthy controls and patients with IDACP (Supplemental Figure 6). The CCs of apoAII-ATQ, apoAII-AT, and apoAII-ATQ/AT were −0.00344, −0.10138, and −0.22291, respectively. There was no significant correlation between apoAII-isoforms and total bilirubin. These data suggest that apoAII-isoforms are not influenced by acute obstructive disease of the biliary duct.

### Distribution of apoAII-isoforms in patients with pancreatic diseases other than IDACP

We evaluated the distribution of the apoAII-isoforms, CA19–9, and DUAPN-2 in patients who would be considered at risk for pancreatic malignancy, such as those with pancreatic endocrine neoplasms, intraductal papillary mucinous neoplasms (IPMNs), mucinous cystic neoplasms (MCNs), serous cystic neoplasms (SCNs), chronic pancreatitis, and other conditions. A decrease in the concentration of apoAII-ATQ/AT relative to healthy controls was observed with all of the diseases that are considered risk factors for pancreatic malignancy. Statistically significant differences in apoAII-ATQ/AT concentration relative to healthy controls were observed in patients with endocrine neoplasms (*P* = 1.88 × 10^−3^), IPMNs (*P* = 1.94 × 10^−9^), chronic pancreatitis (*P* = 3.22 × 10^−6^), and others (*P* = 2.12 × 10^−2^).

The apoAII-ATQ/AT AUC values for distinguishing patients with various gastroenterologic diseases from healthy controls were 0.84 for endocrine neoplasms, 0.92 for IPMNs, 0.816 for MCNs, 0.983 for SCNs, 0.992 for chronic pancreatitis, and 0.951 for other diseases (Supplemental Table 3). In contrast, CA19–9 AUC values for distinguishing patients with endocrine tumors, IPMNs, MCNs, SCNs, chronic pancreatitis, and other diseases from healthy controls were 0.564, 0.565, 0.545, 0.313, 0.334, and 0.398, respectively. The DUPAN-2 AUC values for distinguishing patients with endocrine tumors, IPMNs, MCNs, SCNs, chronic pancreatitis, and other diseases from healthy controls were 0.587, 0.575, 0.775, 0.437, 0.555, and 0.437, respectively (Supplemental Table 3). ApoAII-ATQ/AT is thus superior to CA19–9 and DUPAN-2 for distinguishing patients at risk for pancreatic malignancy from healthy controls.

### Blind validation for early detection of pancreatic cancer with the reference set from NCI-EDRN

**Figure 5:**
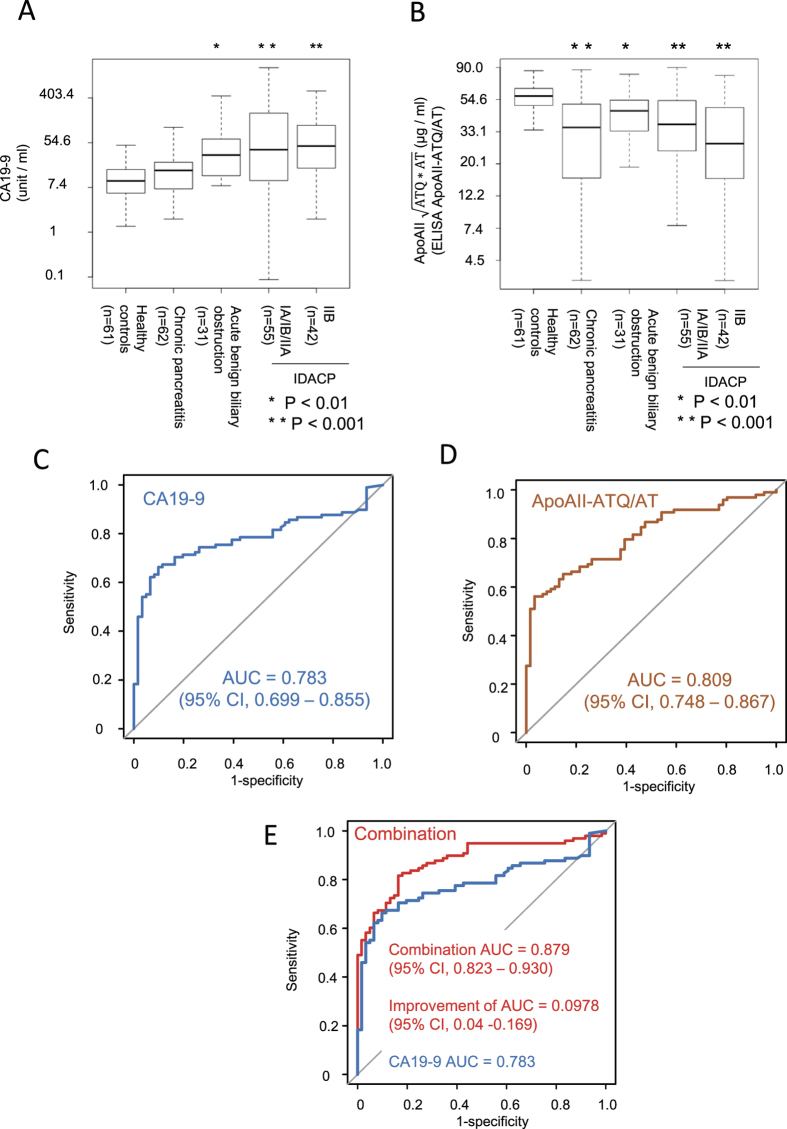
Distribution of CA19–9 and ApoAII-ATQ/AT in the pancreatic cancer reference set of NCI EDRN. Boxplot presentation of the distribution of (log-scale) biomarker of CA19–9 (**A**) and apoAII-ATQ/AT (**B**) values by disease types: healthy controls, subjects with chronic pancreatitis, acute biliary obstruction, early-stage cancer or late-stage cancer. Axes reflect true marker values and outliers are not displayed (Student’s t-test, *p < 0.01, **p < 0.001) ROC curves for single biomarker of (**C**) CA19–9 (blue line) and (**D**) apoAII-ATQ/AT (brown line). (**E**) ROC curves for combination biomarker (red line) with CA19–9 and apoAII-ATQ/AT and for single biomarker of CA19–9 (blue line). Estimated AUCs with 95% confidence intervals (95% CIs), which were obtained by bootstrapping (n = 500) are presented (n = 500).

The EDRN pancreatic reference set, which was comprised of serum samples, was sent blinded to the Japanese team. The team performed the assays and sent the data to the EDRN Data Management and Coordination Center (DMCC) for analysis and determination of the performance of the biomarker(s). Because the Japanese team was blinded to the clinical status of the samples, there was no bias in the analysis. Statistically significant increases in CA19–9 compared to healthy control were observed in acute benign biliary obstruction (Student’s t-test p = 1.67 × 10^−3^), stage-IA/IB/IIA (p = 5.32 × 10^−4^), and stage-IIB (p = 8.21 × 10^−8^) (Fig. 5A). Statistically significant decreases in apoAII-ATQ/AT compared to healthy control were observed in chronic pancreatitis (p = 3.00 × 10^−7^), acute benign biliary obstruction (p = 1.91 × 10^−3^), stage-IA/IB/IIA (p = 8.40 × 10^−7^), and stage-IIB (p = 9.11 × 10^−8^) (Fig. 5B). AUC values of CA19–9 and apoAII-ATQ/AT as single biomarkers to distinguish patients with early stage pancreatic cancer (stage-I/II) were 0.783 [95% confidence interval (95% CI), 0.699–0.855], and 0.809 (95% CI, 0.748–0.867), respectively (Fig. 5C,D). The AUC value of apoAII-ATQ/AT was higher than that of CA19–9. The AUC value based on the linear combination of log-transformed CA19–9 and apoAII-ATQ/AT, estimated from a linear logistic regression model [equation-2, Combination biomarker (linear logistic regression model, CA19–9 and ApoAII-ATQ/AT) = 12.065 + 0.503*log(CA19–9) – 3.403*log(ApoAII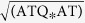], was 0.879 (95% CI, 0.823–0.930). The improvement in AUC of the biomarker combination compared with the single biomarker CA19–9 was 0.0978 (95% CI, 0.040–0.169), which was statistically significant (Fig. 5E).

## Discussion

Here, we demonstrated that the measurement of apoAII-isoforms in plasma enables the detection of the early stages of IDACP and identification of patients at high risk for pancreatic malignancy. We developed ELISAs to measure the plasma concentrations of two apoAII-isoforms, apoAII-ATQ and apoAII-AT. The results obtained using the apoAII-ATQ and apoAII-AT ELISAs can serve as a surrogate biomarker to estimate the amount of apoAII-ATQ/AT.

Over the past decade, a number of researchers have used proteomic techniques to identify biomarkers for detecting the early stages of pancreatic cancer^4^,^5^,^6^,^7^,^8^. In 2005, we identified a plasma biomarker for the detection of the early stages of pancreatic cancer from peak profiles in surface-enhanced laser desorption/ionization time-of-flight (TOF) MS^4^. Although we first identified a significant decrease in a 17,252 m/z plasma protein in patients with IDACP compared with healthy controls, we could not isolate this plasma protein^4^. In 2007, Ehmann *et al.* also reported a reduction in a 17,252 m/z serum protein in patients with IDACP, and they identified it as a unique isoform of apoAII, apoAII-ATQ/AT^7^.

Human apoAII is composed of 77 amino acids and forms a homo-dimer through a disulfide bond involving Cys6^9^. Five apoAII-isoforms distinguishable by molecular weight have been identified to date. It has been reported that the C-terminus of the circulating apoAII homo-dimer is modified^6^,^10^,^11^; however, the mechanism through which this occurs is unclear.

In healthy controls, the distributions of apoAII-ATQ/ATQ, -ATQ/AT, and -AT/AT remained in homeostatic balance. Data for the healthy controls clustered in the two-dimensional scatter graphs. However, in IDACP patients, the distributions of circulating apoAII-isoform homo-dimers were uniquely altered (Supplemental Figure 4). Two different distributions of apoAII-isoforms were observed in samples from IDACP patients, one in which the predominant isoform expressed was apoAII-ATQ/ATQ, which has a higher molecular weight than apoAII-ATQ/AT, and one in which the distribution was characterized by predominant expression of apoAII-AT/AT, -AT/A, or -A/A, which is lower in molecular weight than apoAII-ATQ/AT (Supplemental Figure 4). Notably, expression of apoAII-ATQ/ATQ alone was not observed in healthy controls (hypo-processing pattern). Likewise, healthy controls did not express apoAII-A/A, the isoform with the lowest molecular weight (hyper-processing pattern). The hypo- and hyper-processing patterns are instead characteristic of IDACP or other pancreatic disorders (Fig. 4, Supplemental Figure 3 and Supplemental Figure 4). Our results suggest that the levels of apoAII-ATQ/AT are reduced in patients with IDACP and other pancreatic disorders under conditions of hyper- or hypo-processing.

Carboxypeptidase A is an exopeptidase that cleaves amino acids from the C-terminus of a protein or peptide. Carboxypeptidase A is a digestive enzyme that is primarily synthesized by the pancreas. Matsugi *et al.* reported that the activity of carboxypeptidase A in serum is elevated in patients with IDACP in comparison with non–pancreatic disease patients^12^. The apoAII isoform hyper-processing phenotype might be explained by increased activity of specific exopeptidases released from the pancreas into the serum in patients with IDACP and other pancreatic disorders^13^. However, Matsugi *et al.* also reported that the AUC for carboxypeptidase A in distinguishing pancreatic cancer from non-pancreatic disease was significantly lower than the AUC for CA19–9^12^. These data suggest that the apoAII isoform hypo-processing phenotype is not the result of exopeptidases released from the pancreas into the serum/plasma in IDACP and other pancreatic diseases. It was considered that this phenotype arises via a different mechanism than the hyper-processing phenotype. Hypo-processing might reflect loss of the ability to synthesize exopeptidases in the pancreas.

To measure an absolute quantity of apoAII-ATQ/AT, we first tried to establish the sandwich ELISA using specific antibodies of both anti-apoAII-AT and -apoAII-ATQ. However, this attempt was unsuccessful.

Theoretically, it is considered that the ELISA kit for apoAII-ATQ reacts with native apoAII-ATQ/ATQ and apoAII-ATQ/AT and that apoAII-AT can react with apoAII-ATQ/AT, -AT/AT, and AT/A. However, as Supplemental Table 1 shows, despite the high CC between the results of ELISA-ApoAII-ATQ and MS-ApoAII-ATQ/ATQ (CC = 0.862), correlation was not recognized between the results of ELISA of apoAII-ATQ and MS of apoAII-ATQ/AT (CC = −0.245). The same phenomenon was recognized between ELISA-ApoAII-AT and MS-ApoAII-ATQ/AT (Supplemental Table 1).

These data suggest that ELISA-ApoAII-ATQ has higher affinity for ApoAII-ATQ/ATQ than ApoAII-ATQ/AT. Moreover, ELISA-ApoAII-AT also has a higher affinity for apoAII/AT/AT than apoAII-ATQ/AT and apoAII-AT/A. Thus, ELISA-ApoAII-ATQ is considered to be a surrogate biomarker for amount of apoAII-ATQ/ATQ, and ELISA-ApoAII-AT a surrogate biomarker for apoAII-AT/AT. If the cause of the decrease of apoAII-ATQ/AT is hyper-processing of apoAII-ATQ/AT and apoAII-AT/AT or hypo-processing of apoAII-ATQ/ATQ, a reduction of apoAII-ATQ/AT would be explained by either reductions of apoAII-AT and -ATQ, or an increase of apoAII-ATQ with decreasing apoAII-AT. Thus, we advocate use of the simple equation-1 in the clinical setting.

The AUC values derived from ELISA results, calculated by equation-1 for apoAII-ATQ/AT in this study, were higher than the AUC values derived from MS data. Because the MS-based assay we used does not allow for quantitative apoAII-ATQ/AT measurements, the assay we previously reported could not be standardized. In contrast, the ELISAs can be standardized easily for each sample using GST fusion proteins of the apoAII-isoforms. Therefore, measurement errors in the ELISAs should be smaller than those obtained using the MS-based assay. AUC values for apoAII-ATQ/AT derived from ELISA results for samples from patients with stages-I/II of IDACP in cohort-1 and -2 were >0.9, suggesting that the determination of apoAII-isoforms via ELISA is well suited for discriminating patients with the early stages of IDACP. Although MS-based assays have recently been used for *in vitro diagnostics* in some clinical settings, an ELISA is a more general clinical assay than an MS-based assay^5^. However, equation-1 cannot predict an absolute quantity of apoAII-ATQ/AT. Despite it not absolutely quantifying apoAII-ATQ/AT, equation-1 correlates well with results of MS-ApoAII-ATQ/AT; thus, the results of this formula are a surrogate biomarker for quantifying apoAII-ATQ/AT. To date, no adequate MALDI-MS–based methods for absolute protein quantification have been developed.

We developed ELISAs for the measurement of apoAII-isoforms to expand the options for *in vitro* diagnostics. There are some reports of a significant reduction of the whole amount of apoAII in pancreatic cancer. However, this reduction was slight, and the AUC value was not sufficient for clinical usage. We suggest that the specific isoform of apoAII should be measured to accurately screen patients with pancreatic cancer and diseases that increase its risk.

CA19–9 is a tumor marker measured in the clinical management of pancreatic cancer. However, CA19–9 is not overexpressed in the early stages of pancreatic cancer^14^. Moreover, CA19–9 expression is not elevated in patients who lack the Lewis antigen, even if they have a large pancreatic tumor^15^. Therefore, the guidelines of the American Society of Clinical Oncology do not recommend the measurement of CA19–9 as a screening test for cancer, particularly pancreatic cancer^16^. In the present study, AUC values for apoAII-ATQ/AT in patients with stage-I IDACP were higher than values for CA19–9 and DUPAN-2. In order to more sensitively detect the early stages of IDACP, we examined a combination assay based on CA19–9 and the apoAII-isoforms. The combination assay enabled detection of stage-I and stage-II IDACP with high sensitivity (95.4%) and specificity (98.3%) (Fig. 3). In addition, the apoAII isoform ELISA enabled the identification of 31 of 36 patients with IDACP in which no increase in CA19–9 was observed. It is possible that measurement of the apoAII-isoforms could play a supportive role in *in vitro* diagnostics, compensating for shortcomings associated with measurement of CA19–9 in clinical situations.

A specific decrease in the level of apoAII-ATQ/AT was not limited to IDACP. Significant decreases in apoAII-ATQ/AT levels were also observed in samples from patients with other gastroenterologic diseases. However, the most drastic decreases in the levels of apoAII-ATQ or -AT were associated with only IDACP and pancreatic diseases, suggesting that pancreatic diseases could be discriminated from other diseases based on the two-dimensional distribution of apoAII-ATQ and -AT levels.

ApoAII-isoforms cannot be used to distinguish IDACP from other pancreatic diseases. Alterations in the distribution of apoAII-isoforms are associated with not only IDACP but also other pancreatic disorders, such as endocrine tumors of the pancreas, IPMN, MCN, SCN, and chronic pancreatitis. Patients with neuroendocrine tumors of the pancreas, IPMN, MCN, and chronic pancreatitis are considered to be at high risk for developing pancreatic malignancy. If patients without symptoms associated with risk factors for pancreatic malignancy can be distinguished from healthy individuals using a non-invasive assay, those at highest risk of pancreatic malignancy can be identified, and pancreatic cancer might be detected at an earlier stage than is currently possible^2^,^17^. The existing pancreatic cancer biomarkers CA19–9 and DUPAN-2 did not identify patients at high risk for pancreatic cancer in the present study. It is possible that apoAII-isoforms are better biomarkers for identifying those at high risk for pancreatic malignancy than CA19–9 and DUPAN-2.

To elucidate clinical applicability, we validated the potential of apoAII-ATQ/AT to detect early pancreatic cancer with the pancreatic reference set from NCI EDRN. Ninety-eight cases with pancreatic cancer were included in the reference set, which was collected to accelerate the clinical applicability of early detection biomarkers of pancreatic cancer. The clinical utility of apoAII-ATQ/AT in comparison with CA19–9 for early detection of pancreatic cancer was demonstrated with the pancreatic cancer reference set. The clinical utility of the combination of CA19–9 and apoAII-ATQ/AT for early detection of pancreatic cancer was demonstrated by both the Japanese cohorts and the pancreatic reference set of EDRN. Although we could validate the clinical benefit of apoAll-ATQ/AT to be superior to CA19–9 to distinguish patients with stage-I/II of IDACP from healthy controls using the EDRN reference set, the AUC value of the EDRN reference set was lower than that of the Japanese cohorts (cohort-1 and cohort-2). This difference in AUC value might be due to several reasons. First, the EDRN pancreatic reference set was comprised of serum samples, while the Japanese cohorts (cohort-1 and cohort-2) were composed of plasma samples. The plasma samples might be better than the serum samples for measuring apoAII-isoforms with ELISA to distinguish patients with early stage of IDACP. Another possible reason is racial variation in apoAll-isoforms. Almost all of the samples of Japanese cohorts are composed of Japanese samples. In contrast, the EDRN reference set included some ethnic groups. The expression level of apoAII-isoforms might be different between Japanese and US people. A difference between the two cohorts is also observed in CA19–9: The AUC values of CA19–9 for the Japanese cohort-2 in stage-I and stage-II to distinguish IDACP from healthy controls were 0.834 and 0.952, respectively. These values were also higher than the AUC value of CA19–9 in the EDRN reference set (0.783) as well. Finally, the difference in AUC estimates between the Japanese and the EDRN cohorts could be attributed to sampling error. The estimated AUC for ApoAII-ATQ/AT in the EDRN cohort was 0.809. The half-width of the 95% CI (0.748–0.867) was 0.06. The variability of the AUC estimate for the Japanese cohort is even larger given the smaller sample size of the stage I/II pancreatic cancers in the Japanese cohort compared to the EDRN cohort. As a result, there is large variability associated with the observed difference in AUC between the two cohorts given the current samples sizes of the two studies. In order to further understand and identify the cause of the discrepancy between the EDRN reference set and the Japanese cohorts, more detailed investigation by international collaboration would be necessary.

Overlaps between IDACP and healthy controls or other diseases in apoAII-isoforms are recognized; however, overlaps are also recognized with CA19–9 and DUPAN2, which are existing biomarkers for IDACP. Pancreatic cancer has a very poor prognosis. If apoAII-ATQ/AT has the potential to detect its early stages and those at risk of disease, this may be an important step in decreasing the mortality of pancreatic cancer. Next, we will plan an experimental screening program using this biomarker in patients with pancreatic cancer.

## Methods

### Ethics statement

This research on human subjects was approved by the National Cancer Center Review Board (20–003, 21–140 and 2014–101) and by the Human Tissue Samples Ethics Committee for R&D, Toray Industries Inc. (HC2013–11, HC2013–126, HC2014–14 and HC2014–38). Written informed consent was obtained from each participant. All experiments were performed in accordance with relevant guidelines and regulations.

### Patient samples

Plasma samples were collected at medical institutions in Japan from patients in two cohorts (namely, cohorts -1 and -2) involving a total of 904 patients (Table 1 and supplemental table 3)^4^,^5^,^6^. Identical sample collection and storage conditions (such as collection tubes, anticoagulation procedures, temperature, and number of freeze/thaw cycles) were employed for plasma samples from patients within the same cohort. All samples were analyzed blindly and randomly by ELISA and MS-based assay.

The study protocol was reviewed and approved by the ethics committee of each participating institution, and informed consent was obtained from all patients. Cohort-1 comprised 261 sex- and age-matched healthy controls (n = 131) and patients with histologically proven IDACP (n = 130). Plasma samples were collected at the NCCH (Tokyo, Japan) between 2002 and 2004, as reported previously (Table 1)^4^,^5^. The IDACP of cohort-1 were staged according to the Fifth edition of General Rules for the Study of Pancreatic Cancer (Japanese Pancreas Society). Cohort-2 comprised 643 patients. Samples of plasma were collected at seven medical institutions in Japan (NCCH, Osaka National Hospital [Osaka, Japan], Jichi Medical School Hospital [Tochigi, Japan], Osaka Medical Center for Cancer and Cardiovascular Disease [Osaka Japan], Tokyo Medical University Hospital [Tokyo, Japan], Osaka Medical College Hospital [Osaka, Japan], and Fukuoka University Hospital [Fukuoka, Japan]) between 2006 and 2008 under and according to Standard Operating Procedures (SOP)^5^. Documentation detailing standard operating procedures was distributed to each institute, and all plasma and serum samples were collected under identical conditions^5^,^6^. Information regarding the final diagnosis or classification of the study subjects (gastroenterologic disease, including IDACP, or healthy control) was collected separately from plasma samples. The plasma samples of cohort-2 were comprised of healthy controls (n = 87), and patients with IDACP (n = 155), pancreatic disease other than IDACP (n = 57), cholangiocacrinoma (n = 26), duodenal carcinoma (n = 11), hepatocellular carcinoma (n = 12), esophageal carcinoma (n = 11), gastric carcinoma (n = 142), and colorectal carcinoma (n = 142). The IDACP of cohort-2 were staged according to the International Union against Cancer (UICC).

EDRN (http://edrn.nci.nih.gov/resources/sample-reference-sets) recently established a reference set for pancreatic cancer. The Pancreatic cancer reference set is comprised of serum samples consented and samples collected prospectively. The specimens were collected under rigorous standards (Pepe *et al.* and Feng *et al.*) at multiple institutions and comprised the patient populations most relevant to the clinical requirements. The control subjects included both healthy people and patients with benign pancreatic conditions. The reference set also includes early-stage disease, which is the most difficult to detect. The reference set contains samples from subjects with pancreatic cancer (stage-I/II n = 98), chronic pancreatitis (n = 62), acute benign biliary obstruction (n = 31), and healthy controls (n = 61)^18^. The pancreatic adenocarcinomas of EDRN were staged according to the criteria in the American Joint Committee on Cancer (AJCC) Staging Manual 7th edition.

The reference set is provided blinded to investigators and the resulting data are analyzed by an independent statistician of the EDRN Data Management and Coordination Center (DMCC) for determination of the performance of the biomarker(s). This allows an unbiased evaluation of candidate biomarkers for pancreatic cancer and enables comparisons between candidate biomarkers as well as the combined use of different biomarkers^19^.

### MS-based assay bead-based chromatography

Plasma samples (50 μl) were desalted/concentrated in quadruplicate by chromatography over hydrophobic C8-coated magnetic beads (Bruker Daltonics, Bremen, Germany). After washing, bound proteins were eluted from the beads by the addition of 10 μl of 80% isopropanol (Wako, Osaka, Japan). The eluate was mixed (1:10, v/v) with α-cyano-4-hydroxycinnamic acid saturated in 0.1% trifluoroacetic acid/50% acetonitrile in H_2_O as the matrix (Wako).

### Quantitative MALDI-MS

Quadruplicate desalted/concentrated samples from each patient were spotted in quadruplicate (0.8 μl/spot) on 16 random positions of a disposable 384-well MALDI plate and then dried. Quantitative MS data for plasma/serum proteins were obtained using an oMALDI-QqTOF-MS instrument (prOTOF 2000; PerkinElmer, Boston, MA). MS settings were as described previously^5^. Peaks were detected and visualized using Mass Navigator software (Mitsui Knowledge Industry, Tokyo, Japan) as previously described^5^.

### Establishment of antibodies and ELISA development

Peptides containing the C-terminal apoAII sequence (CRRRVNFLSYFVELGTQPATQ, peptide 1) or those of the processed form (CRRRVNFLSYFVELGTQPAT, peptide 2) were conjugated at the N-terminal cysteine residue to either ovalbumin using maleimide-activated ovalbumin or to keyhole limpet hemocyanin. Female BALB/C mice were immunized with peptide 1 to establish the anti-apoAII-ATQ antibody. Spleen cells from the immunized mice were fused with mouse myeloma SP-2 cells, and the standard hybridoma technique was adopted^20^,^21^. The hybridoma supernatant was first screened by ELISA using peptide 1, then negative screening was done using peptide 2.

Rabbits were immunized with peptide 2 to establish the anti-apoAII-AT antibody. The antiserum was incubated with peptide 2-conjugated resin to enable adsorption of the antibodies. Affinity chromatography with peptide 1 was used to minimize the potential for non-specific adsorption. ELISAs were carried out according to the method of Mike *et al.*^22^. The study protocol was reviewed by the Animal Care and Use Committee and approved by the head of the test facility (Approval No.: AC2011–131 and OE2014–9), and performed in accordance with the Guidelines for Animal Experiments, Research & Development Division, Toray Industries, Inc.

### Measurement of apoAII-isoforms with ELISA

The plasma or serum samples were diluted to 1/5000 to measure apoAII-AT with ELISA apoAII-AT and ELISA apoAII-ATQ kits. The antigens were captured and detected using methods described above. Following an enzymatic reaction with the substrate for peroxidase (HRP Microwell Substrate One component; SurModics, Inc, Edina, MN) at room temperature for 30 min, the absorbance at 450 nm was measured. The whole amount of apoAII was measured by using the APOA2 (human) ELISA kit (Abnova, Taipei, Taiwan) according to the manufacturer’s instructions.

### Measurement of CA19-9 and DUPAN-2

Serum samples from patients in cohort-2 were analyzed using commercially available ELISA kits for CA19–9 (Lumipulse Prestro CA19–9, Fujirebio Inc., Tokyo, Japan) and DUPAN-2 (Detamina Dupan-2, Kyowa Medix Co., Ltd., Tokyo, Japan).

### Statistical analyses

The significance of differences was determined using either Student’s t-test or the chi-square test^23^,^24^. ROC curves were generated and AUC values were calculated using R-project (http://www.r-project.org/)^5^.

To identify biomarker combinations for separating cases from controls, a linear logistic regression model was fitted on individual log-transformed biomarkers. Empirical estimates of the ROC curve and the area under the ROC curve (AUC) were then calculated based on the predicted risk score. In addition, 95% bootstrap percentile confidence intervals for AUC estimates were constructed, based on 500 bootstrap replicates obtained by resampling stratified on case/control status.

## Additional Information

**How to cite this article**: Honda, K. *et al.* Plasma biomarker for detection of early stage pancreatic cancer and risk factors for pancreatic malignancy using antibodies for apolipoprotein-AII isoforms. *Sci. Rep.* **5**, 15921; doi: 10.1038/srep15921 (2015).

## Supplementary Material

Supplementary Information

## Figures and Tables

**Table 1:** Clinicopathologic characteristics of the 261 cases in cohort-1.

	Healthycontrolsn = 130	IDACPn = 131	P-value
Age			
Mean	62.5	61.8	0.566*
SD	10.8	9.01	
Sex			
Male	68	72	0.667**
Female	62	59	
Clinical stage***			
I		2	
II		10	
III		12	
IV		102	
Unknown		5	
Tumor location			
Head of pancreas		57	
Body or tail		66	
Unknown		8	

IDACP, invasive ductal adenocarcinoma of the pancreas; SD, standard deviation.

^*^Student’s t-test.

^**^Fisher’s exact test.

^***^Fifth edition of General Rules for the Study of Pancreatic Cancer (Japanese Pancreas Society).

**Table 2:** Concentration of apoAII-ATQ/AT as determined by ELISA at each clinical stage of IDACP in cohort 2.

	Number	Average(μg/ml)	SD	P-value*	AUC
Healthy control	87	66.7	12.7		
IDACP stage**					
I	6	38.8	17.2	**9.92** × **10**^**−3**^	0.939
II	35	36.7	13.3	**2.85** × **10**^**−15**^	0.957
III	36	36.8	18.3	**4.02** × **10**^**−13**^	0.926
IV	78	36.4	14.9	**5.71** × **10**^**−30**^	0.946

SD, standard deviation; AUC, area under the curve; IDACP, invasive ductal adenocarcinoma of the pancreas.

^*^Student’s t-test. Bold indicates statistically significant difference.

^**^The International Union against Cancer (UICC).
