# Supplementary material for: Plasma biomarker for detection of early stage pancreatic cancer and risk factors for pancreatic malignancy using antibodies for apolipoprotein-AII isoforms
Source: Sci Rep. 2015 Nov 9;5:15921. doi: 10.1038/srep15921 (PMC4637825; doi:10.1038/srep15921)
Supplement: Supplementary Information [file srep15921-s1.pdf]

Plasma biomarker for detection of early stage pancreatic cancer and risk factors for pancreatic malignancy using antibodies for apolipoprotein-AII isoforms

Kazufumi Honda[1, 13], Michimoto Kobayashi[2], Takuji Okusaka[3], Jo Ann Rinaudo[4] Ying Huang[5], Tracey Marsh[5] Mitsuaki Sanada[2], Yoshiyuki Sasajima[2], Shoji Nakamori[6], Masashi Shimahara[7], Takaaki Ueno[7], Akihiko Tsuchida[8], Naohiro Sata[9],Tatsuya Ioka[10], Yohichi Yasunami[11], Tomoo Kosuge[12], Nami Miura[1], Masahiro Kamita[1], Takako Sakamoto[1], Hirokazu Shoji[1] Giman Jung [2], Sudhir Srivastava[4], Tesshi Yamada[1]

1 Division of Chemotherapy and Clinical Research, National Cancer Center Research Institute, Tokyo 104-0045, Japan.

2 Toray Industries, Inc., New Frontiers Research Labs, Kanagawa 248-8555, Japan.

3 Hepatobiliary and Pancreatic Oncology Division, National Cancer Center Hospital, Tokyo 104-0045, Japan.

4 National Cancer Institute, Division of Cancer Prevention, Rockville, MD 20852, USA

5 Public Health Sciences Division, Fred Hutchinson Cancer Research Center, Seattle 98109-1024, WA

6 Department of Surgery, Osaka National Hospital, National Hospital Organization, Osaka 540-0006, Japan.

7 Department of Oral Surgery, Osaka Medical College, Osaka 569-8686, Japan.

8 Department of Gastrointestinal and Pediatric Surgery, Tokyo Medical University, Tokyo 160-0023, Japan.

9 Department of Surgery, Jichi Medical University, Tochigi 329-0498, Japan.

10 Department of Hepatobiliary and Pancreatic Oncology, Osaka Medical Center for Cancer and Cardiovascular Diseases, Osaka 537-0025, Japan.

11 Department of Regenerative Medicine and Transplantation, Fukuoka University Faculty of Medicine, Fukuoka 814-0018, Japan.

12 Hepatobiliary and Pancreatic Surgery Division, National Cancer Center Hospital, Tokyo 104-0045, Japan.

13, Japan Agency for Medical Research and Development (AMED) CREST, Tokyo 100-0004, Japan

Correspondence: Professor K. Honda, 5-1-1 Tsukiji Chuoku, Tokyo 104-0045,

Japan. E-mail: khonda@ncc.go.jp Tel: +81-3-3542-2511

Supplemental information.

Supplemental figure 1-6, and supplemental table 1-3.

Supplemental figure 1

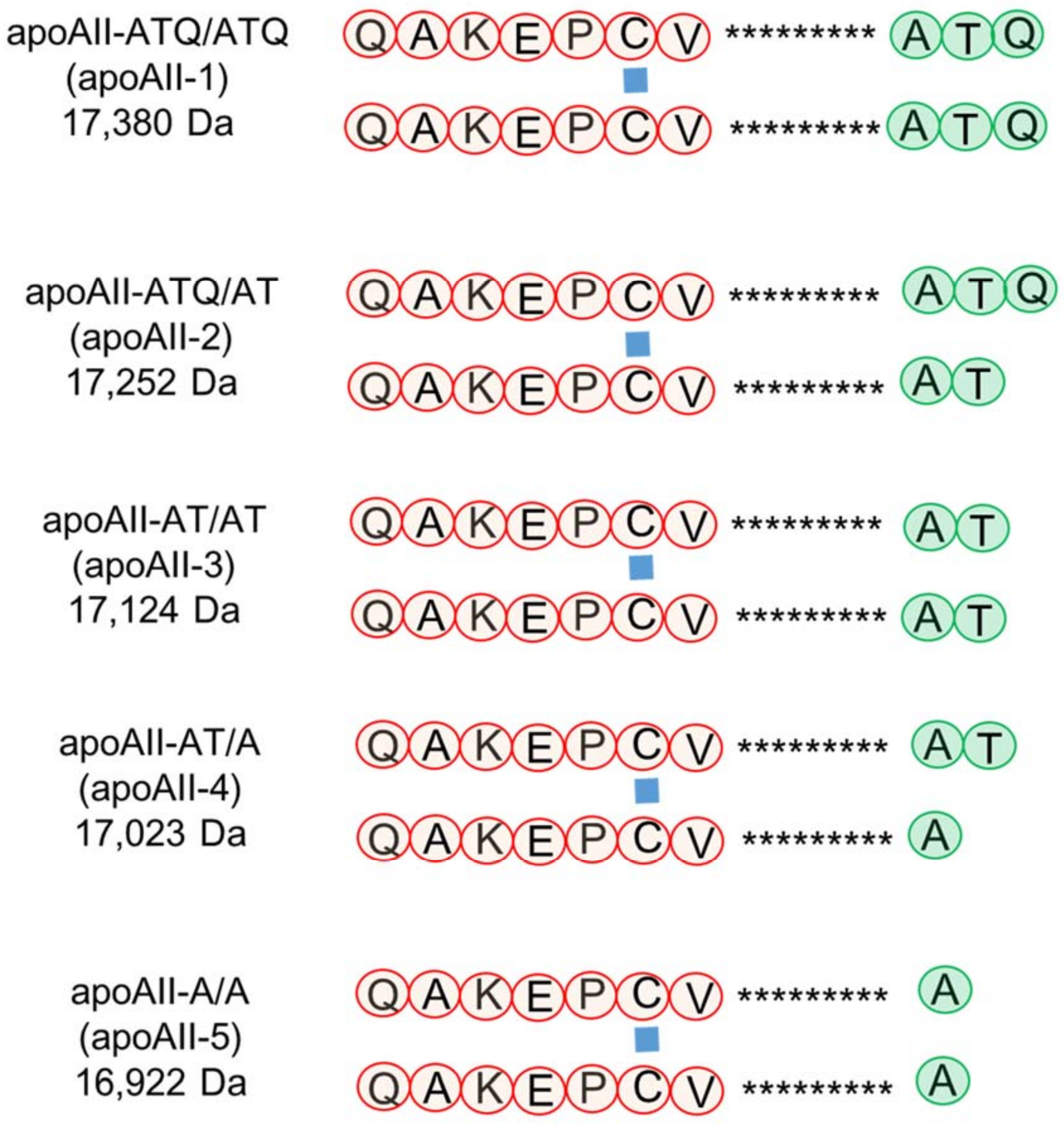

Supplemental figure 1. Amino acid sequences of the apoAII isoforms.

ApoAII is comprised of 77 amino acids and forms a homo-dimer in humans via a disulfide bond involving Cys6. The theoretical molecular weights of the five isoforms of apoAII are shown at left.

Supplemental figure 2

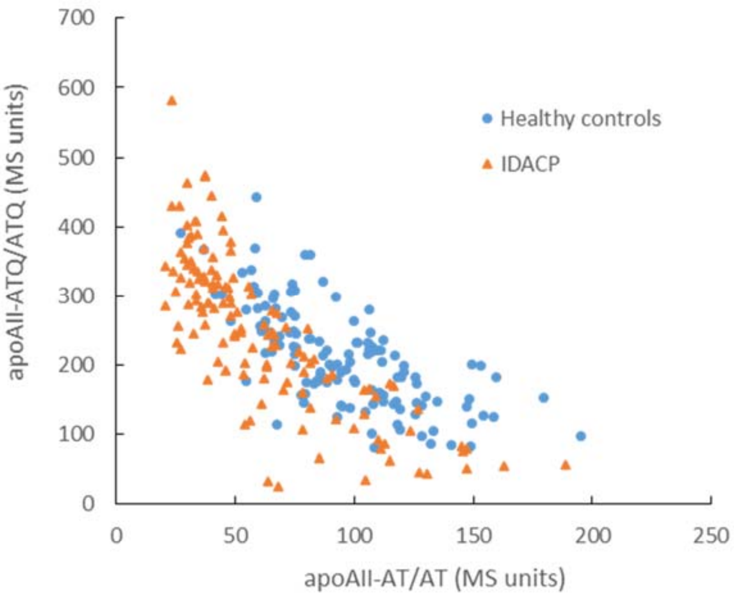

Supplemental figure 2. Two-dimensional scatter graphs apoAII-ATQ/ATQ and apoAII-AT/AT.

Levels of apoAII-ATQ/ATQ and –AT/AT were measured by MALDI-MS, and the distribution of apoAII-ATQ/ATQ and apoAII-AT/AT are shown as two-dimensional scatter graphs. Blue circles, healthy controls; orange triangles, IDACP.

Supplemental figure 3

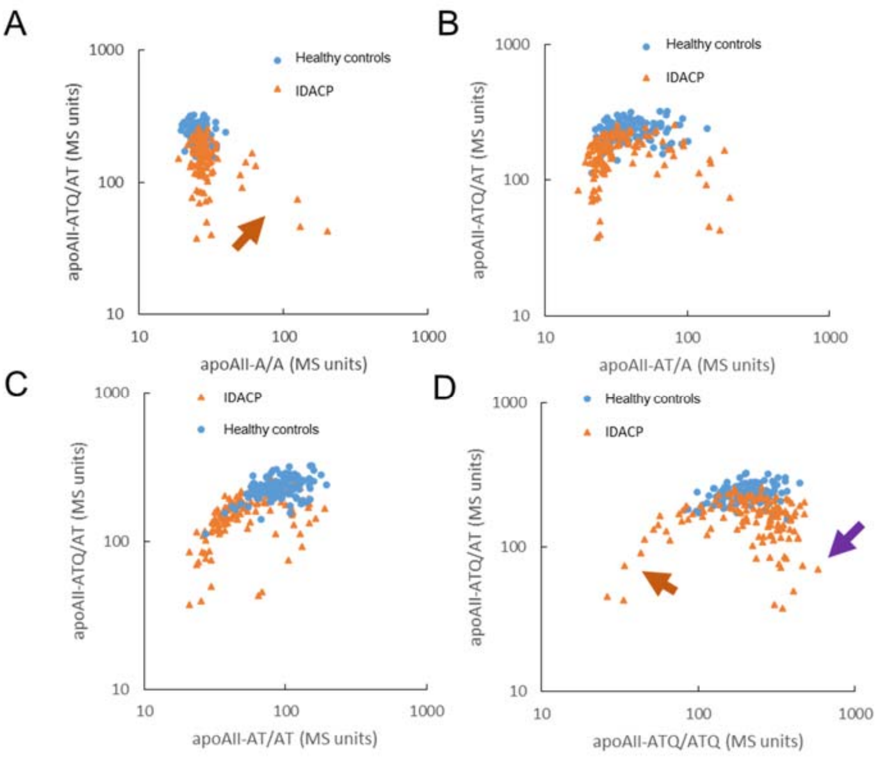

Supplemental figure 3. Two-dimensional scatter graphs of apoAII-ATQ/AT and other isoforms.

Levels of the five apoAII isoforms were measured by MALDI-MS, and the distribution of apoAII-ATQ/AT and other isoforms are shown as two-dimensional scatter graphs (A-D). (A) apoAII-ATQ/AT and -A/A, (B) apoAII-ATQ/AT and -AT/A, (C) apoAII-ATQ/AT and -AT/AT, and (D) apoAII ATQ/AT and -ATQ/ATQ. Blue circles, healthy controls; orange triangles, IDACP. Purple arrow, representative hypo-processing pattern; brown arrows, representative hyper-processing pattern.

Supplemental figure 4

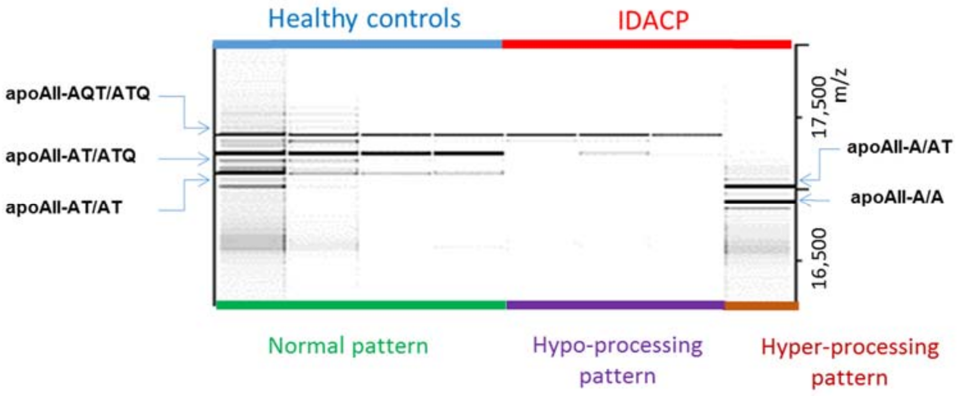

Supplemental figure 4. Representative gel mobility image of MS spectra of normal, hypo-processing, and hyper-processing patterns.

MALDI-MS spectra were converted to gel-mobility images (16,500-17,500 *m/z*). The expression patterns were different in each case. Top blue, healthy controls; Top red, IDACP; bottom green, normal pattern; bottom purple, hypo-processing pattern; bottom brown, hyper-processing pattern.

Supplemental figure 5

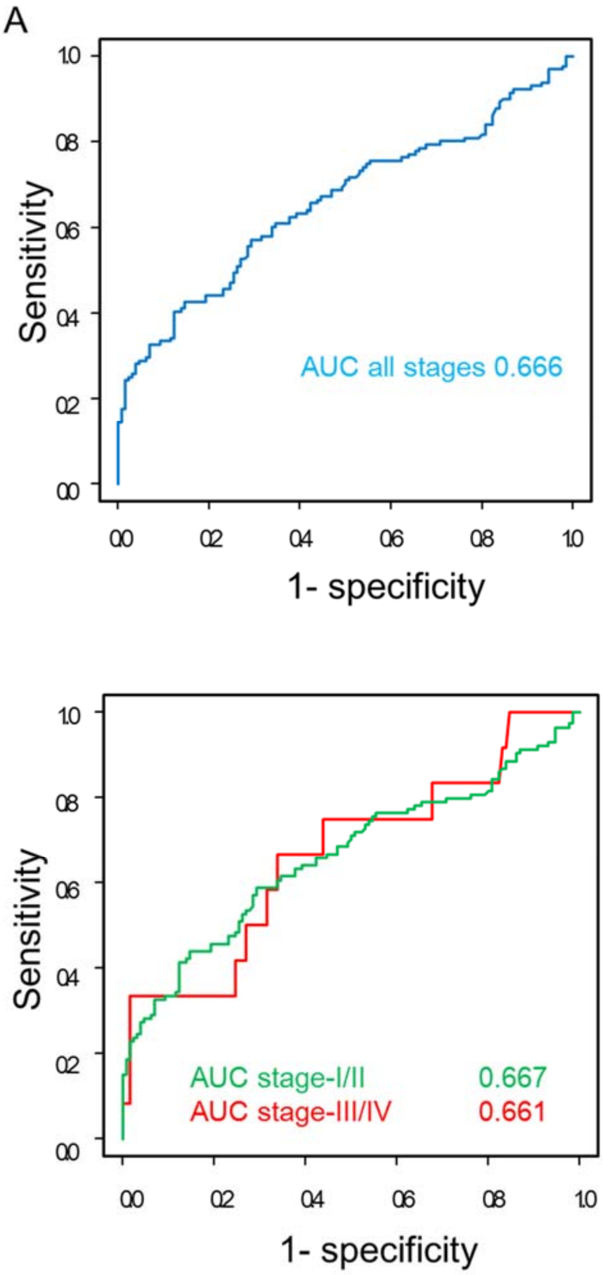

Supplemental Figure 5, ROC analysis for the whole amount of apoAII.

ROC analysis to distinguish the patients with IDACP of all stages from healthy controls (blue line, AUC = 0.666) (A). ROC analyses to distinguish the patients with stage-I/II (green line, AUC = 0.667) or stage-III/IV (red line AUC = 0.661) IDACP from healthy controls.

Supplemental figure 6

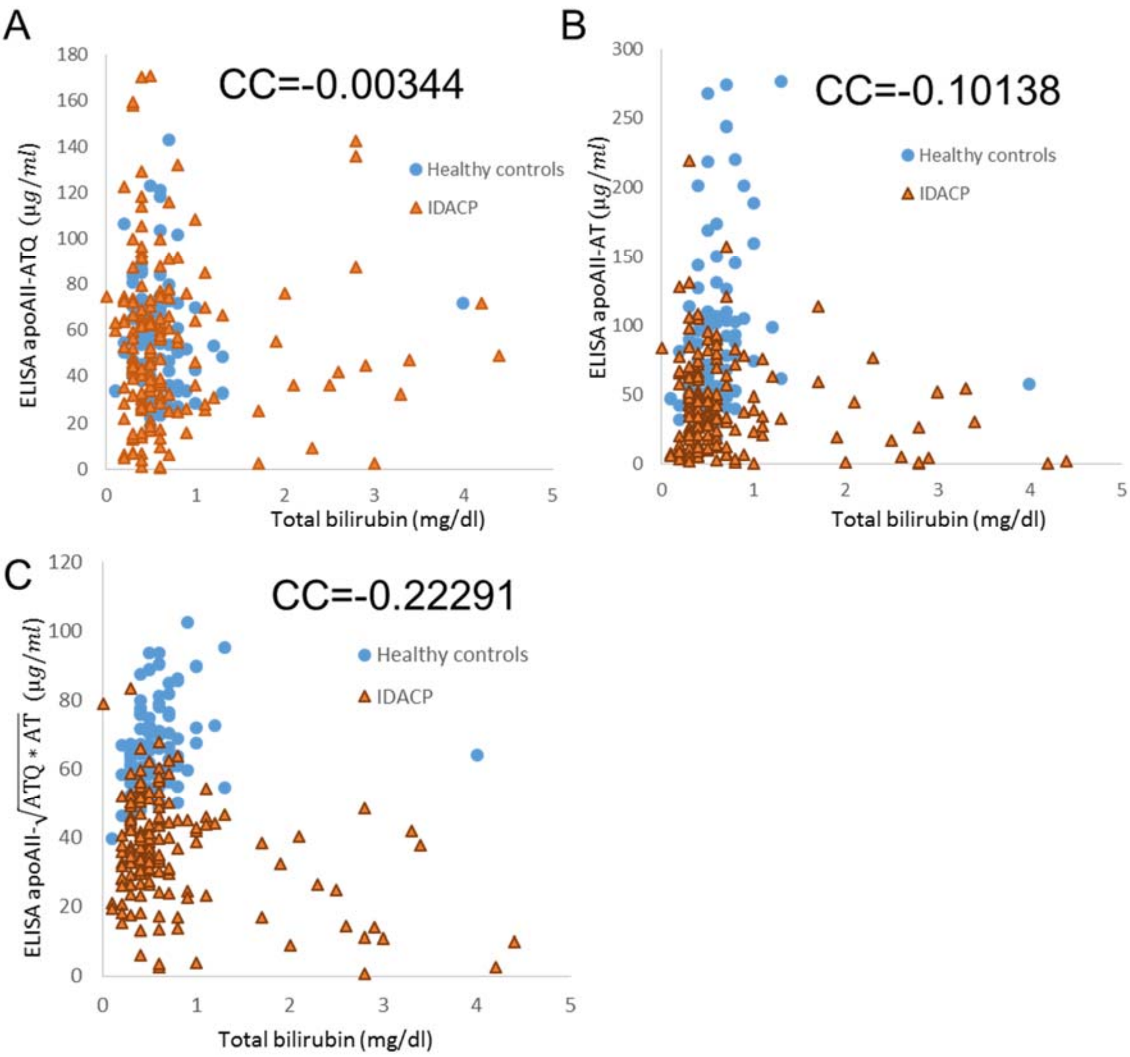

Supplemental Figure 6. Correlation between concentration of apoAII-isoforms and total bilirubin in plasma.

Scatter graphs of concentration of ELISA apoAII-ATQ (A), ELISA apoAII-ATQ (B), and ELISA apoAII-$\sqrt{\mathrm{ATQ} * \mathrm{AT}}$ (C). Blue circles, healthy controls; brown triangles,

IDACP. Correlation coefficients (CC) between concentration of apoAII-isoforms and total bilirubin were calculated from the results of healthy controls and patients with IDACP

Supplemental table 1. The correlation between the measurements for apoAII-isoforms with ELISA and MALDI-MS

		MALDI-MS measurement				
		ApoAII-A/A	ApoAII-AT/A	ApoAII-AT/AT	ApoAII-ATQ/AT	ApoAII-ATQ/ATQ
ELISA measurement	ApoAII-ATQ	-0.233	-0.570	-0.670	-0.245	**0.862**
	ApoAII-AT	-0.010	0.502	**0.831**	0.502	-0.580
	ApoAII √(ATQ/AT)	-0.302	-0.033	0.480	**0.824**	-0.132

ELISA, enzyme-linked immunosorbent assay; MALDI, matrix-assisted laser desorption/ionization; apoAII, apolipoprotein-AII. Bold values show correlations greater than 0.8

Supplemental Table 2. Distributions of apoAII isoforms in healthy controls and patients with various gastroenterologic malignancies and benign diseases.

	Number of patients	ELISA-apoAII-ATQ/AT			ELISA-apoAII-ATQ			ELISA-apoAII-AT		
		Average (μg/ml)	SD	P-value*	Average (μg/ml)	SD	P-value*	Average (μg/ml)	SD	P-value*
Healthy controls	87	66.7	12.7		58.3	25.5		94.4	58.6	
IDACP	155	36.6	17.4	**5.09×10^{-39}**	54.1	35.9	0.288	42.6	34.6	**9.64×10^{-12}**
Pancreatic disease other than IDACP	57	38.5	16.8	**2.22×10^{-18}**	56.3	46.3	0.764	73.5	97.9	0.151
Cholangiocarcinoma	26	43.3	18.8	**1.16×10^{-6}**	49.5	39.6	0.294	58.8	49.2	**3.28×10^{-3}**
Duodenal carcinoma	11	37.7	17.5	**2.17×10^{-4}**	47.8	32.7	0.327	43.7	25.3	**2.16×10^{-5}**
Hepatocellular carcinoma	12	39.9	14.7	**3.97×10^{-5}**	86.4	52.5	0.095	28.9	25.3	**1.22×10^{-7}**
Esophageal carcinoma	11	48	7.09	**4.71×10^{-7}**	63.4	31.9	0.623	48.3	28.6	**2.65×10^{-4}**
Gastric carcinoma	142	53.8	18.3	**2.12×10^{-9}**	43.7	21.8	**1.72×10^{-5}**	86.9	68.8	0.379
Colorectal carcinoma	142	50.4	16.1	**3.98×10^{-15}**	48.5	22.1	**3.59×10^{-3}**	67.6	48.1	**4.41×10^{-4}**

apoAII, apolipoprotein-AII; IDACP, invasive ductal adenocarcinoma of the pancreas; SD, standard deviation. *P-values calculated using the Student's t-test.

Supplemental Table 3. Distributions of apoAII-ATQ/AT, CA19-9, and DUPAN-2 as determined by ELISA in patients with pancreatic diseases other than IDACP in cohort-2.

	Number of patients	ApoAII $\sqrt{(ATQ * AT}$				CA19-9				DUPAN-2			
		Average (μg/ml)	SD	AUC	P-value*	Average (units/ml)	SD	AUC	P-value*	Average (units/ml)	SD	AUC	P-value*
Healthy controls	87	66.7	12.7			11.6	11.3			27.1	12.0		
Endocrine neoplasms	11	41.6	20.0	**0.84**	**1.88×10^{-3}**	16.5	16.8	0.564	0.364	43.5	36.7	0.587	0.170
IPMNs	26	40.5	15.3	**0.92**	**1.94×10^{-9}**	15.1	15.2	0.565	0.278	51.4	94.0	0.575	0.192
MCNs	5	50.0	13.9	**0.816**	0.0526	221.5	468.9	0.545	0.373	683.8	762.1	0.775	0.126
SCNs	2	41.4	8.2	**0.983**	0.128	5.7	2.5	0.313	0.107	24.0	0.0	0.437	**1.86×10^{-2}**
Chronic pancreatitis	9	23.1	13.1	**0.992**	**3.22×10^{-6}**	6.7	8.2	0.334	0.130	35.2	28.5	0.555	0.420
Others	5	35.7	14.5	**0.951**	**2.12×10^{-2}**	7.5	5.5	0.398	0.235	24.0	0.0	0.437	**1.86×10^{-2}**

ApoAII, apolipoprotein-AII; SD, standard deviation; AUC, area under the curve, bold >0.80. IPMNs, intraductal papillary mucinous neoplasms; MCNs, mucinous cystic neoplasms; SCNs, serous cystic neoplasms. *P-values were caluculated using Student's t-test, bold <0.05.
